# Foliar application of melatonin improve the number of secondary branches and secondary branch grains quality of rice

**DOI:** 10.1371/journal.pone.0307368

**Published:** 2024-08-20

**Authors:** Feiyu Yan, Guoliang Zhang, Hongliang Zhao, Zhiwei Huang, Yuan Niu, Mingchao Zhu

**Affiliations:** 1 School of Life Sciences and Food Engineering, Huaiyin Institute of Technology, Huai’an, Jiangsu, China; 2 Seed Science and Technology Research Center, Huaiyin Agricultural Institute of Xuhuai Prefecture, Huai’an, Jiangsu, China; 3 Crop Breeding Research Office, Jiangsu Tianfeng Seed Industry Company Limited, Huai’an, Jiangsu, China; University of Florida Institute of Food and Agricultural Sciences, UNITED STATES OF AMERICA

## Abstract

Melatonin plays an important role in plant growth and development. However, little information is available about melatonin regulating rice panicle structure and yield. This study explored the regulatory effects and mechanisms of melatonin spraying before the panicle differentiation stage on rice panicle structure and grain quality. The results showed that spraying melatonin before panicle differentiation increased rice yield, which was mainly reflected in the increase in spikelets per panicle and the percentage of filled grains. In addition, melatonin treatment significantly increased the panicle length. The results of panicle structure analysis showed that the increase in spikelets per panicle caused by melatonin was attributed to the significant increase in the number of secondary branches, total number of secondary branch spikelets, and number of spikelets per secondary branch. The results showed that melatonin can increase the content of zeatin, auxin, and gibberellin, and reduce the content of abscisic acid. These results showed that melatonin affected panicle structure by regulating hormone content, thereby improving yield. In addition, melatonin improves the processing quality, appearance quality, and nutritional quality of secondary branch grains. The above results indicate that application of melatonin improves the number of secondary branches and the quality of grainss on secondary branches.

## 1 Introduction

According to the current population growth rate, the World Health Organization predicts that the world population will reach 9 billion by 2050 [1]. Food is the primary necessity for people. Feeding up the growing population is an urgent problem that needs to be solved. As food rations for more than half of the world’s population, rice (*Oryza sativa* L.) supplies 19% of the world’s daily per capita calorie consumption [2]. The global consumption of milled rice is predicted to reach 590 million tons by 2040 [3]. Therefore, a high yield is a primary goal for rice cultivation. Limited by limited land resources, it is difficult to expand the rice planting area, and increasing yield per unit area has always been the common goal of rice researchers.

Rice yield is determined by three main yield components: panicle number, spikelet number per panicle, and 1,000-grain weight, and is affected by development and environment [4]. The main rachis, rachis branches (including primary and secondary branches), and spikelets are the main components of the rice panicles. The spikelet is the basic unit of the inflorescence attached to the branch by the pedicel, and the branch is generated from the node of the main rachis [5]. In the research and practice of rice breeding, spikelet number is considered the key characteristic to improve yield. Panicle structure, including panicle length, shape, and number of primary and secondary branches, is an important factor in determining the spikelet number per panicle [2]. Extensive studies have shown that the number of primary and secondary branches is significantly positively correlated with the number of spikelets per panicle [6].

In addition to yield, the quality of rice grains is also receiving increasing attention. Improving grain quality while ensuring high yield is also beneficial for ensuring that more and more rice consumers accept it [7, 8]. The quality of rice includes processing quality, appearance quality, nutritional quality, and taste quality. The flowering time of spikelets in different growing parts of rice panicles is not synchronized, which can lead to asynchronous grain filling and development. Due to the close correlation between rice quality traits and grain filling process, there are differences in grain quality at different positions on the same panicle [9]. In general, the spikelets on the first branch have early flowering, fast grain filling initiation, and short grain filling time, while the spikelets on the second branch of the rice panicle have late flowering, slow grain filling initiation, and long grain filling time, which leads to poor grain quality on the second branch [10].

Plant hormones are the main internal factors that regulate branch development [2]. Plant hormones such as cytokinin (CTK), auxin, gibberellin (GA), abscisic acid (ABA), methyl jasmonate, and brassinolide participate in the regulation of panicle structure, forming a complex regulatory network to coordinate the development of yield-related traits, thus controlling the yield potential of plants. CTK is an evolutionarily conserved regulator of plant cell division and meristem activity, and plays a key role in flower organ development. The level of CTK in the inflorescence meristem is positively related to the number of flower organs by promoting meristem activity [11]. Auxin is considered a negative regulator of inflorescence meristem activity. The dynamic outflow of auxin is crucial for axillary meristem establishment [12]. Changes in the polar transport and distribution of auxin can improve the number of spikelets per panicle and the plant structure [13]. GA plays an important role in the regulation of cell division and elongation in plant vegetative organs [14]. Crosstalk between GA and CTK is related to the regulation of spikelets per panicle. GA oxidase reduces the accumulation of GA and increases the CTK level in the spikelet meristem, leading to an increase in spikelets per panicle [15]. ABA and ethylene negatively regulate spikelet development and grain filling after anthesis. The lower ratio of ABA to ethylene and ACC content in inferior spikelets is directly related to the cell division rate, grain filling rate, and the resulting grain weight [16].

Melatonin is a multifunctional molecule that is found in plants. It is involved in the regulation of plant growth, development of aerial organs, root morphology, floral transition, and other processes. It is also involved in regulating many biological and abiotic stress responses [17, 18]. It also interacts with plant hormones. In tomatoes, melatonin enhances the expression of auxin signaling genes *IAA19*, *IAA24*, *PIN1*, *PIN3* and *PIN7* [19]. Exogenous melatonin promotes the upward regulation of ABA catabolism genes and GA biosynthesis genes and the downward regulation of the ABA biosynthesis gene, which endows cucumber with higher salt tolerance [20]. Similarly, melatonin treatment can upregulate the expression of CTK biosynthesis genes and signal response transcription factors and downwardly regulate abscisic acid-related biosynthesis and signaling genes, thus alleviating the heat damage of ryegrass [21].

In recent years, the regulatory effects of melatonin on crop yield and grain quality have received attention. Studies have shown that soaking seeds with melatonin can improve tomato yield. Similarly, the fruit weight of the melatonin-treated pears increased by 47.85%. Inhibition of melatonin synthesis-related genes leads to a decrease in rice yield [22]. The melatonin synthesis gene *OsCOMT* has been proven to be a positive regulator of rice yield [23]. Recent research has shown that melatonin can regulate the carbon and nitrogen metabolism of rice, thereby improving yield [24]. Melatonin has also been reported to improve rice yield by regulating the antioxidant capacity and metabolites [25]. However, melatonin-rich transgenic rice expressing sheep serotonin N-acetyltransferase delays flowering and reduces yield by 33% [26]. Many studies have described the mechanism by which melatonin regulates the yield of rice and other crops, but these studies have focused on gene functions and physiological processes [24, 27, 28]. There are also a few studies indicating that melatonin can alleviate the adverse effects of high temperature on rice grain quality, mainly due to its regulation of starch synthesis [7].

Panicle structure and spikelet number per panicle are important factors that affect rice yield and grain quality. There is no relevant research on the regulation of melatonin in the structure of rice panicles and the number of spikelets per panicle. This study used melatonin pretreatment before the panicle differentiation stage of two rice varieties to measure the hormone content in different stages of rice panicle differentiation. The panicle structure and yield during the mature stage, as well as the quality of grains on different branches, were also measured to expand the understanding of the role and mechanism of melatonin in regulating rice yield and quality.

## 2 Materials and methods

### 2.1 Plant materials and experimental design

This experiment was conducted during the rice-growing season of 2022 (May-October) at the experimental farm of the Huaiyin Institute of Technology, Huai’an City, China. In this study, two conventional japonica rice varieties, Nanjing 9108 (NJ9108) and Nanjing 9308 (NJ9308), were selected as experimental materials. The soil type is sandy loam soil with 22.6 g/kg organic matter, 115.7 mg/kg alkali hydrolyzable N, 27.6 mg/kg Olsen-P, and 82.5 mg/kg exchangeable K. The sowing date was May 12, and the seedlings were transplanted at a density of 12 cm × 30 cm on June 12. The amount of base fertilizer was 25 kg compound fertilizer (N: P: K = 15:15:15), tillering fertilizer was 20 kg urea, and panicle fertilizer was 20 kg compound fertilizer (N: P: K = 30:0:5). During the entire growth period of rice, the field management measures were consistent, and diseases, insects, and weeds were strictly controlled.

Melatonin treatment was performed before young panicle differentiation, combined with the leaf age remainder method and microscopic examination to determine the accurate spraying time. The concentration of melatonin (M5250-5G, Sigma-Aldrich (Shanghai), China) sprayed was 200 μM/L, and the dosage was 100 ml/m^2^, spraying for two consecutive days at nightfall. The area of the experimental plot was 100 m^2^ and three replicates were set for each variety.

### 2.2 Sampling

During transplantation, 300 main stems (100 plants in each replication) were marked. Combined with microscopic examination and leaf age remainder method, samples were taken at key points of the panicle differentiation stage for later hormone content determination, including bract differentiation stage (I), branching differentiation stage (II), spikelet differentiation stage (III), pollen mother cell meiosis (IV) and pollen filling (V). At maturity, three consecutive rice clusters were randomly selected for yield composition analysis.

### 2.3 Panicle structural

Thirty main stems were collected on the day of harvest. The panicle length was measured using a centimeter ruler. The number of primary branches, secondary branches, primary branch spikelets, and secondary branch spikelets were recorded. The primary branch grain filling percentage, secondary branch grain filled percentage, weight of the primary branch 1,000-grain weight, and secondary branch 1,000-grain weight were calculated. The particle density was then calculated.

### 2.4 Grain yield and yield components

The yield components were determined from plants in a one square meter area randomly sampled from each plot. The percentage of filled grains is defined as the percentage of filled grains (total spikelets). After harvesting 300g of seed samples, they were naturally dried to a moisture content of 14% and stored at 4°C for quality determination.

### 2.5 Plant hormone content

The samples collected in the field were quickly frozen in liquid nitrogen and brought back to the laboratory for fine segmentation to retain the young panicles. An enzyme-linked immunosorbent assay kit (JXBY1871/JXBY1594/JXBY2943, Jianxin Technology Co., Ltd, Nanjing, China; YJ077235, Warbio Technology Co., Ltd, Nanjing, China) was used to determine the hormone (zeatins (ZRs), indole acetic acid (IAA), GA, and ABA) content. The specific operation steps followed the manufacturer’s instructions.

### 2.6 Grain quality

Analyze the quality traits of rice in accordance with the Chinese national standard (GB/T 17891–2017). The processing quality is monitored using hullers and rice mills, and the results are expressed as weight percentages. Use a grain appearance analyzer to measure length, width, chalkiness rate, and chalkiness. Measure protein content and amylose content using a grain analyzer. After protein extraction and separation [29], the content of albumin, globulin, gliadin, and glutelin was determined using the Coomassie Brilliant Blue method.

### 2.7 Statistical analysis

All data measurements were repeated thrice. SPSS (version 22.0; SPSS Inc., Chicago, IL, USA) was used for analysis of variance. When p<0.05, the mean value was determined using Duncan’s method. The path analysis was completed using SPSS22.0. The correlation test between indicators was completed using R (3.6.1 The R Foundation for Statistical Computing). All drawings were performed using GraphPad Prism (GraphPad Software Inc., San Diego, CA, USA).

## 3 Results

### 3.1 Melatonin treatment increased rice yield

**Fig 1:**
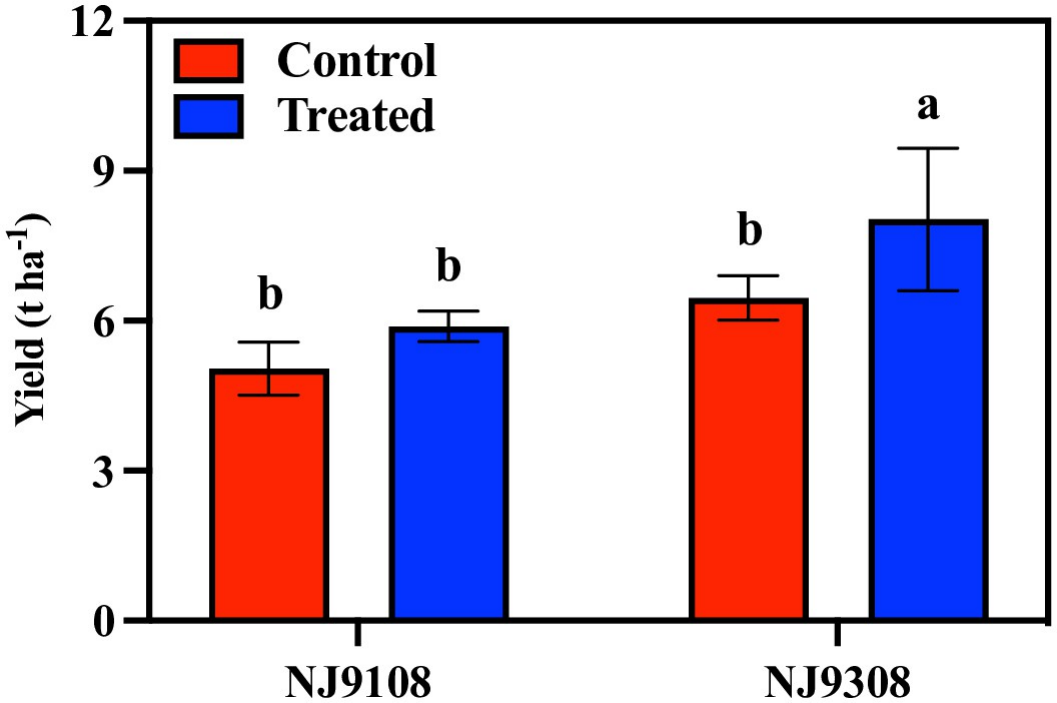
Effect of melatonin spraying on the yield of NJ9108 and NJ9308. Vertical bars represent the ±standard errors of the mean (n = 3) where these exceed the size of the symbol. Different letters above the columns indicate significant differences at the p < 0.05 level within the same index.

The application of melatonin at the panicle differentiation stage increased rice yield. For strain NJ9108, melatonin treatment increased the yield by 16.72%. For NJ9308, melatonin treatment significantly increased the rice yield by 24.42% (Fig 1).

### 3.2 The increase of rice yield after melatonin treatment benefited from higher spikelets per panicle

**Fig 2:**
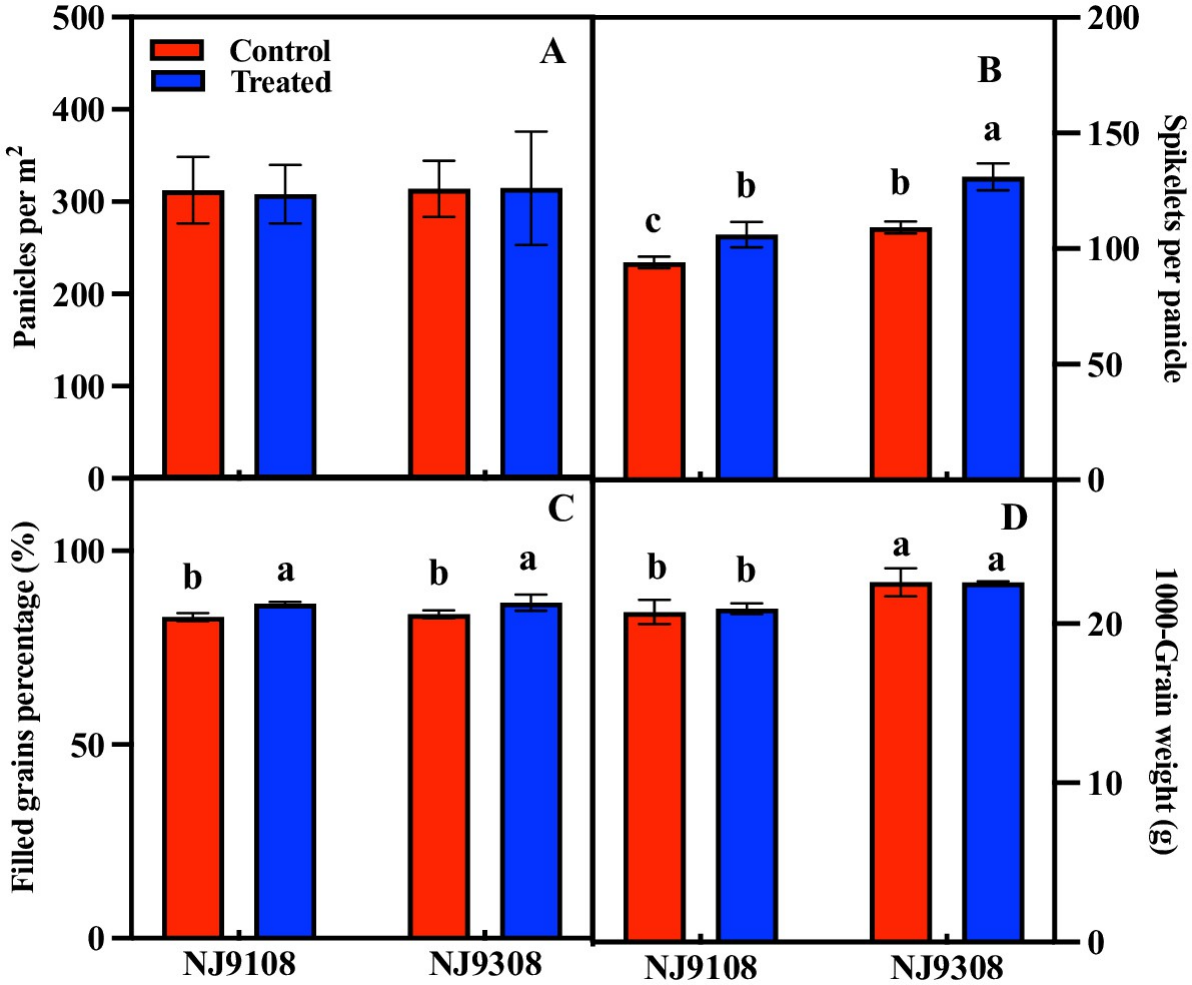
Effect of melatonin spraying at on yield components of NJ9108 and NJ9308. (A) Panicle per m^2^; (B) Spikelets per panicle; (C) Filled grains percentage; (D) 1000-grain weight. Vertical bars represent the ±standard errors of the mean (n = 3) where these exceed the size of the symbol. Different letters above the columns indicate significant differences at the p < 0.05 level within the same index.

The results of yield components showed that melatonin significantly increased the percentage of filled grains and spikelets per panicle of the two rice varieties but had no significant effect on the panicles per square meter and 1,000-grain weight (Fig 2). The results of the path analysis showed that for rice without melatonin treatment, the highest contribution to rice yield came from panicles per square meter. However, for rice with melatonin treatment, the highest contribution to rice yield came from spikes per square meter (Table 1).

**Table 1:** Path analysis of yield and yield components of rice varieties with different treatments.

Treatment	Yield components	Path coefficient (direct action)	Indirect path coefficient (indirect action)				
			Panicles per m^2^	Spikelets per panicle	Filled grains	1000-Grain weight	Total
Control	Panicles per m^2^	0.625		-0.021	0.045	-0.139	-0.115
	Spikelets per panicle	0.506	-0.026		0.020	0.299	0.294
	Filled grains	0.068	0.414	0.149		-0.033	0.531
	1000-Grain weight	0.412	-0.211	0.367	-0.005		0.151
Treated	Panicles per m^2^	0.659		-0.180	0.171	0.002	-0.007
	Spikelets per panicle	0.803	-0.148		-0.035	-0.036	-0.218
	Filled grains	0.201	0.560	-0.138		-0.002	0.420
	1000-Grain weight	-0.037	-0.028	0.772	0.013		0.757

### 3.3 Higher spikelets per panicle of rice after melatonin treatment come from the increase of secondary branches

**Fig 3:**
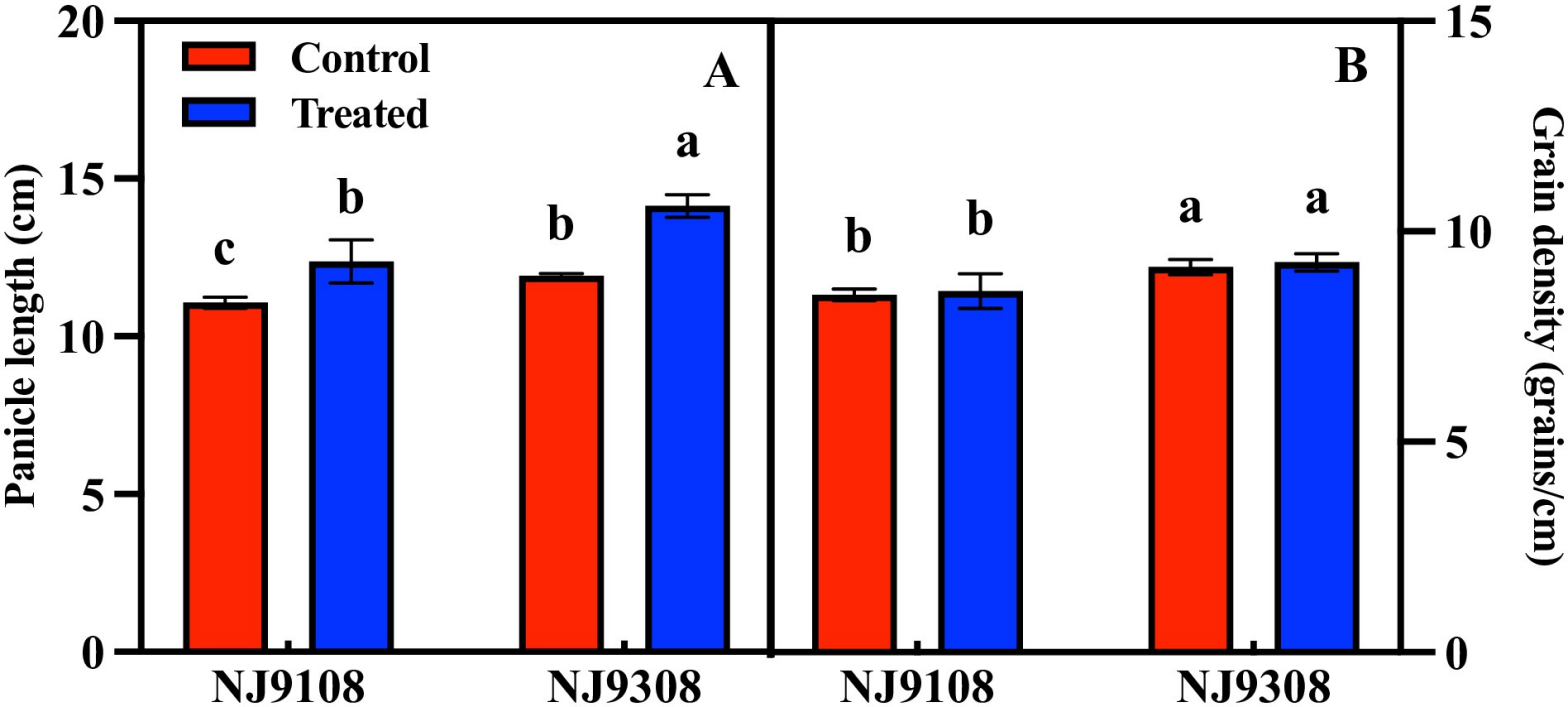
Effect of melatonin spraying on panicle length and grain density of NJ9108 and NJ9308. (A) Panicle length; (B)Grain density. Vertical bars represent the ±standard errors of the mean (n = 3) where these exceed the size of the symbol. Different letters above the columns indicate significant differences at the p < 0.05 level within the same index.

The panicle length of the two rice varieties increased significantly after melatonin treatment, but the grain density was not significantly affected by melatonin treatment (Fig 3). The grain density of NJ 9308 is significantly higher than that of NJ 9108.

**Fig 4:**
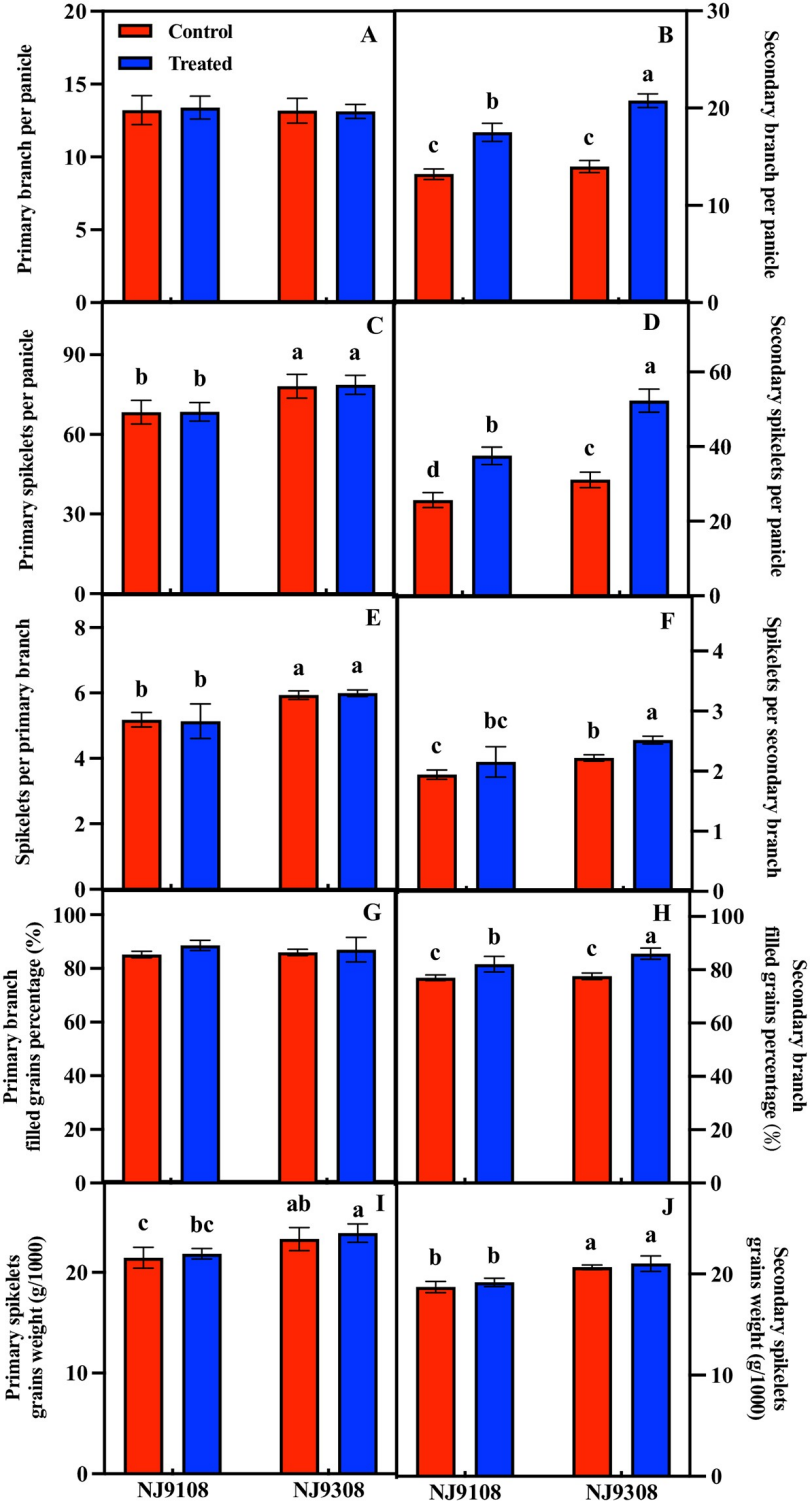
Effect of melatonin spraying on NJ9108 and NJ9308 panicle structure. (A) Primary branch per panicle; (B) Secondary branch per panicle; (C) Primary spikelets per panicle; (D) Secondary spikelets per panicle; (E) Spikelets per primary branch; (F) Spikelets per secondary branch; (G) Primary branch filled grains percentage; (H) Secondary branch filled grains percentage; (I) Primary spikelets grains weight; (J) Secondary spikelets grains weight. Vertical bars represent the ±standard errors of the mean (n = 3) where these exceed the size of the symbol. Different letters above the columns indicate significant differences at the p < 0.05 level within the same index.

Melatonin treatment significantly increased the number of secondary branches per panicle, number of secondary spikelets per panicle, number of spikelets per secondary branch, and filled grains of secondary branches but had no significant effect on the 1000-grain weight of secondary branches. Melatonin treatment did not have a significant regulatory effect on primary branch-related indicators (Fig 4).

**Fig 5:**
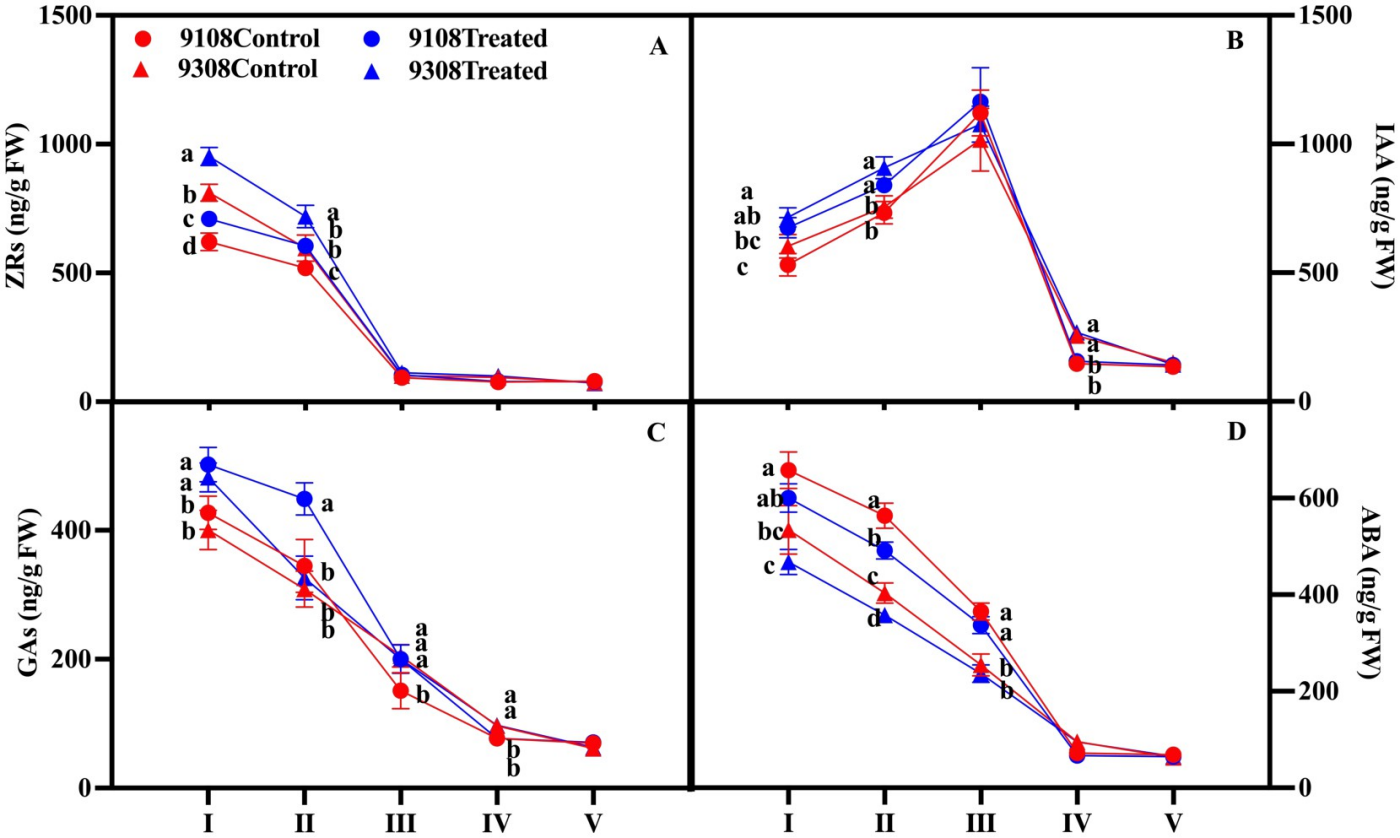
Effect of melatonin spraying on hormone content in rice panicle during panicle differentiation. (A) ZRs; (B) IAA; (C) GAs; (D) ABA. (I) Bract differentiation stage; (II) branching differentiation stage; (III) spikelet differentiation stage; (IV) pollen mother cell meiosis; (V) pollen filling. Vertical bars represent the ±standard errors of the mean (n = 3) where these exceed the size of the symbol. The different letters above each stage of panicle differentiation indicate significant differences at the p < 0.05 level within the same index.

Melatonin treatment significantly increased the ZRs content of young panicles during the first and second stages of panicle differentiation. The IAA in the 1st, 2nd, and 4th stages significantly increased. The GA in the 1st, 2nd, 3rd and 4th stages was significantly increased. The ABA content of the 1st, 2nd, and 3rd stages significantly decreased, and the two varieties showed the same performance (Fig 5).

### 3.4 Melatonin treatment improved the quality of secondary branch grains

Melatonin significantly increased the brown rice rate, milled rice rate, and head milled rice rate of secondary branch grains, and the performance of the two varieties was consistent. However, melatonin did not show a regulatory effect on the processing quality of seeds on primary branches (Table 2).

**Table 2:** Processing quality of rice grains on different branches and stems after melatonin treatment.

Treatment	Branches	Brown Rice Rate (%)		Milled Rice Rate (%)		Head Milled Rice Rate (%)	
		NJ9108	NJ9308	NJ9108	NJ9308	NJ9108	NJ9308
CK	Primary	84.03 ab	84.20 a	74.90 a	75.54a	70.61 a	70.46 a
	Secondary	81.01 c	80.32 c	70.96 c	69.94c	61.99 c	60.75 b
MT	Primary	84.34 a	84.39 a	75.84 a	76.11a	70.36 a	70.07 a
	Secondary	82.97 b	82.75 b	73.38 b	73.07b	64.70 b	62.20 b

Different letters indicate statistical significance at the 0.05 probability level.

Melatonin significantly increased the length of grains on primary and secondary branches, but did not affect grain width. Melatonin treatment significantly reduced the chalkiness rate and degree of secondary branch grains (Table 3).

**Table 3:** Appearance quality of rice grains on different branches and stems after melatonin treatment.

Treatment	Branches	Grain Length (mm)		Grain Width (mm)		Chalkiness Rate (%)		Chalkiness Degree (%)	
		NJ9108	NJ9308	NJ9108	NJ9308	NJ9108	NJ9308	NJ9108	NJ9308
CK	Primary	4.38 b	4.35 b	2.59	2.53	23.13 b	24.25 b	5.24 b	5.28 b
	Secondary	4.33 c	4.31 c	2.58	2.54	29.96 a	30.76 a	6.29 a	6.31 a
MT	Primary	4.43 a	4.40 a	2.58	2.54	19.44 b	19.15 c	4.82 b	4.66 b
	Secondary	4.36 b	4.32 c	2.57	2.53	27.45 a	27.89 ab	5.85 a	5.95 a

Different letters indicate statistical significance at the 0.05 probability level.

Melatonin significantly reduced the amylose content and gel consistency of primary and secondary branch grains, with consistent performance in both varieties. However, melatonin did not show a regulatory effect on protein content (Table 4).

**Table 4:** Nutritive quality of rice grains on different branches and stems after melatonin treatment.

Treatment	Branches	Amylose Content (%)		Gel Consistency (mm)		Protein Content (%)	
		NJ9108	NJ9308	NJ9108	NJ9308	NJ9108	NJ9308
CK	Primary	15.95 b	15.61 b	92.26 b	92.67 b	7.12 b	7.08 b
	Secondary	13.73 d	13.68 d	80.88 d	80.92 c	8.82 a	8.18 a
MT	Primary	16.72 a	16.63 a	96.38 a	96.29 a	6.65 b	6.54 b
	Secondary	14.89 c	14.62 c	85.22 c	85.25 d	8.08 a	8.92 a

Different letters indicate statistical significance at the 0.05 probability level.

Melatonin treatment significantly reduced the albumin content in NJ9308 primary and secondary branch grains, as well as the gliadin content in NJ9108 primary and secondary branch grains. Melatonin significantly reduced the content of glutelin in secondary branch grains, and the performance of the two varieties was consistent (Table 5).

**Table 5:** Protein content of rice grains on different branches and stems after melatonin treatment.

Treatment	Branches	Albumin (%)		Globulin (%)		Gliadin (%)		Glutelin (%)	
		NJ9108	NJ9308	NJ9108	NJ9308	NJ9108	NJ9308	NJ9108	NJ9308
CK	Primary	0.33 b	0.35 bc	0.42	0.43 b	0.61 c	0.60 b	4.91 c	5.25 c
	Secondary	0.39 a	0.40 a	0.45	0.46 a	0.75 a	0.72 a	6.43 a	6.58 a
MT	Primary	0.32 b	0.32 c	0.43	0.42 b	0.54 d	0.53 c	5.29 c	4.92 c
	Secondary	0.36 a	0.37 b	0.44	0.45 a	0.68 b	0.67 a	5.91 b	5.98 b

Different letters indicate statistical significance at the 0.05 probability level.

## 4 Discussion

### 4.1 Melatonin improves the yield of rice by increasing spikelets number per panicle

Melatonin has been widely proven to exist in plants, and the exogenous application of melatonin has also been proven to be an effective approach to improve crop tolerance, yield, and quality [30]. In this study, the yield of rice was improved by melatonin pretreatment before panicle differentiation, and the performance of the two rice varieties was consistent (Fig 1). Similarly, improved effects of melatonin on crop yields have been reported in maize, wheat, and soybeans [31–33]. Photosynthesis is the basic physiological process of plant dry matter accumulation and energy acquisition. Melatonin has been shown to regulate the synthesis and degradation of chlorophyll through the transcription of related genes and hormone signals and has a protective effect on photosynthetic proteins [34, 35]. Based on these functions, melatonin provides a material basis for regulating the increase in sugar and starch production in the photosynthetic carbon cycle [36]. Overexpression of the melatonin synthesis gene can also improve rice yield. The number of panicles, grains per panicle, and 1,000-grain weight of rice increased significantly, but the grain-filling rate decreased significantly [23]. Reducing the concentration of melatonin by inhibiting melatonin synthase will lead to the loss of rice yield, which is mainly manifested in the significant decrease in 1,000-grain weight and panicle number, and a significant increase in grain infertility rate [22]. However, the results of our study showed that melatonin increased rice yield by increasing the number of spikelets per panicle and grain filling rate but did not have a regulatory effect on the number of panicles and 1,000-grain weight (Fig 2; Table 1). We speculate that the reason for this difference is the duration of maintenance of endogenous high concentrations of melatonin in rice. Rice overexpressing the melatonin synthesis gene maintained a high endogenous melatonin concentration throughout its growth period. However, in this study, the increase in endogenous melatonin concentration was maintained for a relatively short time by spraying melatonin externally; therefore, the regulatory effect was weak.

### 4.2 Melatonin improves the number of spikelets per spike by increasing the number of secondary branches

The rice panicle structure is a key target for high yield. The number of spikelets per panicle is positively correlated with panicle length, the number of primary branches, and the number of secondary branches [37]. Panicle length determines the number of grains that it can accommodate [38]. Compared with standard varieties, some varieties can achieve high yield through higher panicle length, primary branch number, and secondary branch number, but this is limited to keeping panicle length unchanged and only increasing branches to improve grain yield [39]. In this study, melatonin treatment significantly increased panicle length, and the two varieties showed the same performance; however, melatonin had no significant effect on grain density (Fig 3). Similarly, increasing the panicle length through organic farming can also improve rice yield. In contrast, the yield of *OsIAGLU* transgenic rice decreased because of the shortening of the panicle length [40]. Panicle branching mode, regulated by the number of primary and (especially) secondary branches, directly determines the total grain number [41]. In this study, melatonin treatment significantly increased the number of secondary branches and secondary spikelets per panicle of rice, the number of spikelets on each secondary branch of the rice panicle, and the grain filling rate of the secondary branch (Fig 4). These results show that the site of action of melatonin seems to be a secondary branch, and it has no regulatory effect on the indices related to the primary branch. The number of primary branches is limited by the number of vascular bundles in the panicle neck, whereas the number of vascular bundles is mainly determined by genetic factors. Research shows that increasing the number of vascular bundles did not lead to an increase in the number of primary branches but led to an increase in the number of secondary branches [42]. Spraying sucrose and glucose on rice at the heading stage significantly increased the number of secondary branches and spikelets but had no significant effect on the number of primary branches and spikelets [43]. The melatonin spraying time in our study was before the panicle differentiation stage. At this stage, the vascular bundle of the panicle neck node has developed, and the number has been determined, which may be the reason why the number of primary branches is not regulated. However, the specific reasons for this require further clarification.

### 4.3 Melatonin regulates the number of secondary branches by regulating the hormone content in panicle

Plant hormones are small regulatory molecules that form a complex regulatory network that coordinates the development of yield-related traits, thereby controlling the yield potential of plants. The number of spikelets per spike depends on the period from bract differentiation to pollen mother cell meiosis [44]. The results of this study showed that melatonin pretreatment had a regulatory effect on the panicle hormone content at the panicle differentiation stage, in which melatonin increased the content of ZRs, IAA, and GA, but decreased the content of ABA (Fig 5), and this regulatory effect was mainly manifested in the early stage of panicle differentiation, the two varieties showed the same performance. Studies on the regulation of branch differentiation by hormones have been widely reported. Overexpression of *OsVIL2* inhibits CTK oxidase-related genes and increases the CTK content of rice, thereby increasing the number of primary branches, secondary branches, and grains per panicle [45]. The insertion of *SP3* gene promotes the decomposition of CTK, reduces the biosynthesis of CTK, and significantly reduces the number of secondary branches and spikelets [46]. Overexpression of the rice *PAY1* gene can increase auxin content and the number of secondary branches and grains per panicle, but it has no significant effect on the number of primary branches [47]. By knocking out *DCL3a*, the contents of GA and brassinolide were reduced, and the number of secondary branches was affected; and the yield of rice was reduced [48].

Most ABA studies on ABA focused on grain filling. Studies have shown that ethylene and ABA synergistically and negatively regulate the development of spikelets after anthesis [16]. The regulatory effect of melatonin on plant endogenous hormone levels and their related genes has been well reviewed [49, 50]. Melatonin has a regulatory effect on almost all plant hormones including GA, auxin, CTK, and ABA. Existing melatonin regulation of rice yield is mostly concentrated at the physiological level. Melatonin can improve rice yield by regulating leaf senescence and vascular bundle development [23], alleviating cell oxidation [51], regulating carbon and nitrogen metabolism [24], and other physiological processes. The results of this study showed that the rice yield and yield components were significantly correlated with panicle type and panicle hormone content. Melatonin can regulate the number of secondary branches by regulating the hormone content in the panicle, thereby increasing the number of spikelets per panicle.

### 4.4 Melatonin improves the quality of secondary branches grains

Extensive research has shown that melatonin has a regulatory effect on crop quality. Treating rice with melatonin during the filling period can alleviate the impact of high temperature on key physicochemical properties of rice quality, and optimize the synthesis of branched starch to improve rice quality [7]. The application of melatonin effectively alleviated the damage of drought to wheat grain quality, and reduced the wet gluten content and gluten content of wheat grains [52]. Similarly, melatonin has been reported to improve the quality of pomegranate [53]. The results of this study indicate that melatonin improves the processing quality (Table 2), appearance quality (Table 3), and nutritional quality (Table 4) of rice secondary branch grains, but does not show a consistent regulatory effect on the protein components of the grains (Table 5). The quality of rice grains is a complex comprehensive indicator that includes processing quality, appearance quality, and nutritional quality. Due to the significant differences between grains within the panicle, the overall grain yield and quality are affected differently [54]. The increase in the number of spikelets per panicle in most rice varieties is mainly attributed to additional grains located on secondary branches [55], but grains in these areas often have poor filling, which leads to higher chalkiness rates. In this study, rice treated with melatonin not only showed a higher number of secondary branch spikelets, but also improved grain quality on secondary branches, indicating that secondary branches obtained more storage components [56]. This may be related to the regulation of crop photosynthesis by melatonin. Melatonin has been widely reported to improve crop photosynthesis [36], but the specific regulatory mechanism still needs further research.

## 5 Conclusions

In this study, spraying melatonin on two rice varieties before panicle differentiation increased yield, mainly because melatonin significantly increased the number of spikelets per panicle. Through the analysis of panicle structure, the higher spikelet number per panicle after melatonin treatment comes from more secondary branches and spikelet number per secondary branch, which is mainly caused by melatonin regulating the hormone content of the rice panicles at the panicle differentiation stage. In addition, melatonin treatment also improved the processing quality, appearance quality, and nutritional quality of secondary branch grains. These results show that melatonin is an important factor in increasing the number of spikelets per panicle and grain yield of rice, and the exogenous use of melatonin is an effective technical way to improve rice yield and quality.

## Supporting information

S1 File The raw data involved in this article. (XLSX)
